# Systematic Review of Line-Field Confocal Optical Coherence Tomography for Diagnosing Pre-Malignant and Malignant Keratinocytic Lesions: Optimising the Workflow

**DOI:** 10.3390/diagnostics15212746

**Published:** 2025-10-29

**Authors:** Maria Luísa Santos e Silva Caldeira Marques, Justin Hero, Mary-Ann el-Sharouni, Marta García Bustínduy, Pascale Guitera

**Affiliations:** 1 Sydney Melanoma Diagnostic Centre, Royal Prince Alfred Hospital, Camperdown, NSW 2050, Australia; 2 Melanoma Institute Australia, The University of Sydney, Sydney, NSW 2065, Australia; 3 Department of Dermatology, University Hospital of the Canary Islands, 38320 San Cristóbal de La Laguna, Spain; 4 Faculty of Medicine, University of La Laguna, 38200 San Cristóbal de La Laguna, Spain; 5 Faculty of Medicine and Health, The University of Sydney, Sydney, NSW 2050, Australia

**Keywords:** line-field confocal optical coherence tomography, LC-OCT, keratinocytic, non-invasive imaging, systematic review, dermatology

## Abstract

**Background**: Line-field confocal optical coherence tomography (LC-OCT) is a non-invasive imaging technique providing high-resolution en-face and cross-sectional views of the epidermis and superficial dermis for in vivo characterisation of actinic keratosis (AK), Bowen’s disease (BD) and squamous cell carcinoma (SCC). Despite its promise, standardised imaging protocols are lacking. **Objective**: This systematic review aims to assess the utility of LC-OCT for diagnosing AK, BD and SCC, with particular emphasis on workflow optimisation and protocol standardisation. **Methods**: A systematic literature search was performed using PubMed, Embase, and Scopus databases (January 2018–October 2024). Two reviewers independently screened the records, extracted data and applied the Confidence in the Evidence from Reviews of Qualitative research (CERQual) framework to assess confidence in key findings. **Results**: Eleven studies met the inclusion criteria. LC-OCT reliably identified key histopathological correlates. Across studies, LC-OCT consistently visualised hyperkeratosis, keratinocytic atypia, parakeratosis, and acanthosis, as well as characteristic vascular alterations and dermal remodeling. LC-OCT also demonstrated its capacity to detect invasive features by revealing disruptions in the dermo-epidermal junction and the presence of tumour strands infiltrating the dermis. Multimodal imaging combined with technical optimisations such as minimal probe pressure, paraffin oil coupling, and dermoscopy-guided localisation, substantially improved image resolution and interobserver concordance. **Conclusions**: This systematic review provides a basis for establishing standardised LC-OCT imaging protocols in keratinocytic tumours. While LC-OCT shows promise as a non-invasive diagnostic tool, further multicenter studies are needed to refine imaging workflows and evaluate the integration of artificial intelligence-based analysis to improve diagnostic accuracy and reproducibility.

## 1. Introduction

Line-field confocal optical coherence tomography (LC-OCT) is a novel, non-invasive imaging modality that provides high-resolution en-face and cross-sectional views of the epidermis and superficial dermis. With an axial resolution of approximately 1 μm, LC-OCT surpasses conventional OCT, while achieving greater penetration depth than reflectance confocal microscopy (~500 μm) [1]. It enables visualisation of keratinocytic atypia, architectural disorganisation, and vascular patterns, making it valuable for diagnosing actinic keratosis (AK), Bowen’s disease (BD), and squamous cell carcinoma (SCC) [2,3]. Beyond qualitative assessment, recent studies have quantified basal keratinocyte budding—a surrogate marker of progression risk—using the PRO score (I–III), and convolutional neural network (CNN) algorithms from two German cohorts reproduced expert grading with over 70% concordance, facilitating real-time AK risk stratification [4,5].

Prevalence estimates for AK vary widely, largely due to methodological heterogeneity and reliance on clinical diagnosis, which underestimates true incidence [6]. Australia reports the highest rates (40–60% of white individuals aged ≥ 40 years) [7,8], compared to ~6.5% in the United States and 1.4–23% in European populations [9,10,11]. AK lesions carry a low but non-negligible risk of malignant transformation to SCC (0–0.075% per lesion-year, rising to 0.53% among patients with prior keratinocyte carcinoma) [12]; moreover, patients with AK have a more than sevenfold increased risk of SCC [13]. Key risk factors include immunosuppression [14], male sex, age ≥ 70 years, and fair skin phenotype [15].

BD poses a diagnostic challenge due to its subtle clinical presentation, which often mimics benign dermatoses [16]. Dermoscopy and RCM both lack specificity: features such as an atypical honeycomb pattern, parakeratosis, targetoid dyskeratotic cells, and tortuous papillary vessels are also seen in hyperkeratotic AK, seborrheic keratosis, and SCC [17]; in the pigmented variant, dense dendritic keratinocytes and vertical “buttonhole” capillaries resemble melanoma or pigmented basal cell carcinoma (BCC) [18]. Given that 3–5% of BD cases progress to invasive SCC [17,19], non-invasive techniques with higher diagnostic specificity are needed.

SCC represents a significant public health burden, with high incidence, recurrence rates, and variable metastatic potential (0.1–9.9%), accounting for 75% of non-melanoma skin cancer mortality [20,21]. In 2021, SCC resulted in around 1.9 million new cases (age-standardised incidence rate of 22.38 per 100,000), with the highest incidence in high-income North America and the highest mortality in Australasia. Over the past 30 years, its incidence has increased by approximately 2.06% per year, particularly in East and Central Asia, largely due to prolonged UV exposure [22,23], exacerbated by ozone depletion, urbanisation, industrialisation, and improved detection [24].

Early and accurate diagnosis is essential to improve patient outcomes and reduce healthcare costs [25]. Although histopathology remains the gold standard, its invasiveness, cost, and potential for adverse and cosmetic sequelae highlight the need for non-invasive alternatives [26]. Although LC-OCT has shown promise in diagnosing BCC [27], its application to keratinocytic tumors—AK, BD, and SCC—may be complicated by bleeding and scaling, which can degrade image quality and hinder accurate interpretation.

Moreover, the lack of standardised LC-OCT diagnostic criteria and imaging protocols restricts clinical adoption. Variability in image acquisition, analysis, and interpretation may cause sampling errors or misinterpretation, while excessive imaging can increase procedural time and reduce cost-effectiveness. Standardised protocols and technician training—enabling remote expert review—could enhance accessibility, consistency, and reliability. Additionally, LC-OCT might facilitate monitoring of treatment response and early detection of recurrence [28,29].

Despite growing interest in LC-OCT, current evidence is fragmented across small, heterogeneous studies, and no standardised diagnostic workflow exists for keratinocytic lesions. A systematic synthesis is therefore needed to consolidate diagnostic criteria, harmonise acquisition and interpretation standards, and support clinical implementation.

Therefore, this systematic review aims to comprehensively evaluate the literature on LC-OCT for AK, BD, and SCC, with an emphasis on workflow optimisation. Specifically, it seeks to (i) identify and appraise studies evaluating the diagnostic features of LC-OCT in these lesions; (ii) extract and synthesise data on lesion-specific LC-OCT imaging characteristics; (iii) determine optimal workflows for LC-OCT examinations; and (iv) identify areas requiring protocol standardisation in LC-OCT imaging and analysis.

## 2. Materials and Methods

### 2.1. Literature Search

This systematic review is registered on PROSPERO (CRD420251039143) and was conducted in accordance with the Preferred Reporting Items for Systematic Reviews and Meta-Analyses (PRISMA) guidelines. A comprehensive literature search was performed in PubMed (*n* = 24), Embase and Embase Classic (*n* = 5), and Scopus (*n* = 9) for studies published between 1 January 2018 and 31 October 2024, which was the date of the last search. The search strategy combined the terms “line field confocal optical coherence tomography,” “LC OCT,” “actinic keratosis,” “AK,” “squamous cell carcinoma,” “SCC,” “Bowen’s disease,” “in situ SCC,” “diagnosis,” “imaging,” “protocol,” and “criteria” using Boolean operators (AND, OR, NOT). Only English-language original research articles were eligible. The full search string is provided in Supplementary Table S1, and the PRISMA 2020 Checklist is presented in Supplementary Table S2.

### 2.2. Eligibility Criteria

**Figure 1:**
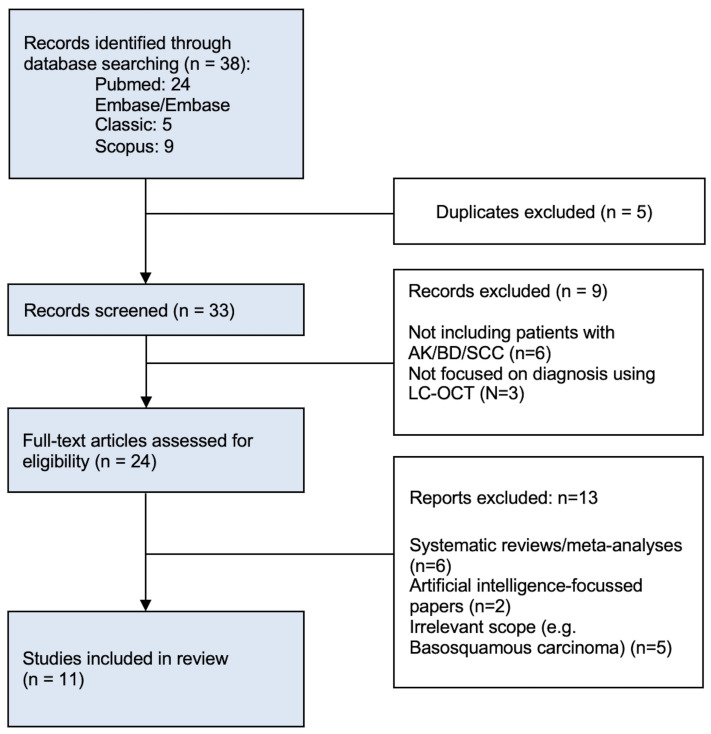
Flow chart of the studies included in the systematic review.

Conference proceedings, pre-prints, reviews, systematic reviews, meta-analyses, and studies focusing on basal cell carcinoma (BCC) or basosquamous carcinoma were excluded. After removing five duplicates, 33 unique records were screened by title and abstract. Records were excluded if they did not include patients with AK, BD, or SCC (*n* = 6) or did not focus on LC-OCT diagnosis (*n* = 3). The remaining 24 full-text articles were assessed for eligibility; thirteen were excluded (six systematic reviews or meta-analyses, two AI-focused studies, and five with irrelevant scope, such as basosquamous carcinoma). AI-based studies were excluded because they primarily report algorithmic performance rather than LC-OCT diagnostic criteria or interpretive features and therefore do not allow extraction of reproducible diagnostic data for clinically applicable synthesis. Eleven studies met the inclusion criteria and were included in the qualitative synthesis (Figure 1).

### 2.3. Data Selection and Extraction

Two independent reviewers (JH, LSC) conducted the screening and selection process; disagreements were resolved by discussion or consultation with a third reviewer (CR). Data extraction used a standardised form to capture study characteristics, lesion types, LC-OCT imaging features, protocols, and limitations. One reviewer (JH) extracted the data, and a second reviewer (LSC) verified all entries; any discrepancies were resolved by consensus. Corresponding authors were contacted for clarification of missing or ambiguous data.

### 2.4. Quality Assessment

The Confidence in the Evidence from Reviews of Qualitative research (CERQual) framework was applied to assess confidence in the synthesised findings, evaluating methodological limitations, coherence, adequacy, and relevance of the data. Given the heterogeneity in study designs and the limited availability of quantitative metrics, a qualitative synthesis approach was chosen. A thematic analysis was then performed to identify recurring patterns in LC-OCT imaging characteristics, workflow recommendations, and interpretive criteria across the included studies.

## 3. Results

Eleven studies met the inclusion criteria and were incorporated into this systematic review. Table 1 summarises the design, sample size, and target conditions of each study, forming the basis for our analysis.

Table 2 presents the CERQual assessment of methodological limitations, coherence, adequacy, and overall confidence. Recommended LC-OCT workflows for AK, BD, and SCC are described in the text, detailing key acquisition procedures and essential technical parameters. The subsequent sections integrate these synthesised findings and discuss their practical applications and clinical relevance for each lesion type.

### 3.1. Key Criteria for Diagnosis

#### 3.1.1. Actinic Keratosis

Multiple studies consistently reported characteristic epidermal changes in AK as visualised by LC-OCT [30,31,32,33,34]. Hyperkeratosis is seen in 42–100% of lesions as irregular hyperreflective areas indicating stratum corneum thickening [28,30,31,32,33,34,35,36,37]. Keratinocytic atypia—evident as variations in cell size, shape and nuclear morphology manifesting as hypo-reflective areas—is present in over 82% of AKs, typically confined to the basal and suprabasal layers [28,30,31,32,34,35,36,37]. Parakeratosis (64–83%) [28,30,31,35,36,37] and acanthosis (approximately 75%) [28,30,31,35,36] are also frequently noted. Finally, LC-OCT may reveal an irregular epidermis with focal tumour budding into the papillary dermis in 45–58% of cases [32,34,35,37].

**Figure 2:**
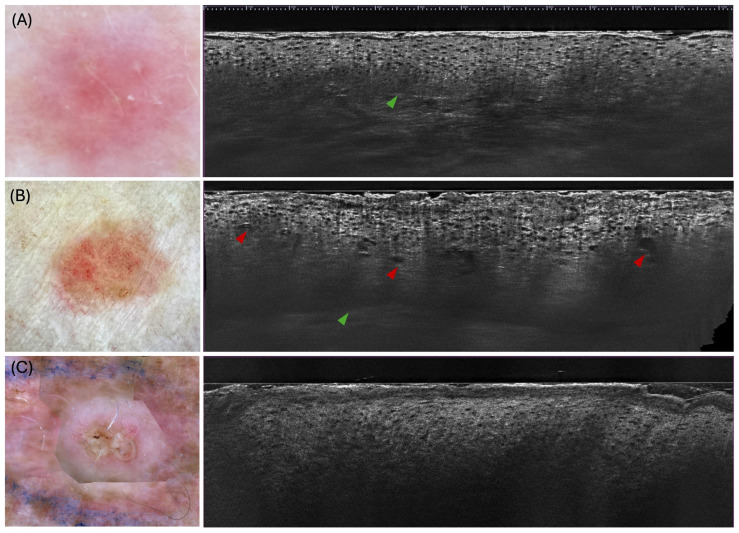
LC-OCT and corresponding dermoscopic images of keratinocytic lesions showing characteristic morphologic features of: (**A**) Actinic keratosis (AK) on the left volar forearm of a 59-year-old woman, with atypical keratinocyte organisation, pleomorphic keratinocyte nuclei, and a well-defined dermo-epidermal junction (DEJ) (green arrows) with budding; (**B**) Bowen’s disease (BD) on the left anterior upper arm of a 68-year-old woman, with hyperkeratosis, pleomorphic keratinocyte nuclei, a well-outlined DEJ (green arrow), and glomerular vessels ascending into the epidermis (red arrows); and (**C**) Squamous cell carcinoma (SCC) on the right anterior upper arm of a 70-year-old man, showing a non-visible DEJ, acanthotic epidermis, hyperkeratosis, and keratinocyte nuclear dysplasia.

The mean epidermal thickness in AK ranged from 105 to 126.2 μm, indicating hyperproliferation [35,36]. The dermo-epidermal junction (DEJ) remains visible in over half of AK lesions, though marked hyperkeratosis can obscure it [30,32,35,36,37,38]. Compared to SCC, AK shows less severe architectural disorganisation [28,30,35,36,38] (Figure 2).

Dilated linear and glomerular vessels have been reported with variable frequency, likely reflecting underlying inflammation and keratinocyte hyperproliferation [30,31,35,36]. Dermal elastosis—visualised on LC-OCT as disrupted collagen fibers and hyporeflective bands within the papillary dermis—has been documented in approximately 26% to 45% of cases [30,32,35,36].

#### 3.1.2. Bowen’s Disease

BD typically presents with marked full-thickness keratinocyte atypia that is more advanced than in AK yet lacks the invasive strands characteristic of invasive SCC [32,35,36] (Figure 2). Hyperkeratosis occurs in 70–100% of lesions, often accompanied by compact parakeratosis in over 60% of cases [35,36]. Epidermal erosion or ulceration is reported inconsistently across series, while moderate acanthosis remains a common finding [32,35,36]. LC-OCT reveals disarranged epidermal architecture and focal tumour budding [35]—reported in approximately 33 to 49% of cases—although these features are generally less pronounced than those observed in invasive SCC [32,36]. The mean epidermal thickness in BD ranged from 141 to 168.5 μm [35,36], which is intermediate between that observed in AK and SCC [32,36]. The DEJ remains well-defined in a highly variable proportion of BD lesions (24–80%) [32,35,36]. A characteristic “bowenoid” pattern—marked by nuclear pleomorphism and prominent nucleoli—may be detected in approximately 90% of BD [36]. Vascular alterations—dilated vessels in 44–70% and glomerular vessels in 20–62% of cases—reflect neoangiogenesis, while dermal elastosis exhibits similarly broad variation across studies [35,36].

#### 3.1.3. Invasive Squamous Cell Carcinoma

In invasive SCC, LC-OCT reveals a spectrum of epidermal and dermal abnormalities far exceeding those seen in AK or BD lesions (Figure 2). Hyperkeratosis is almost universal (70–100%) [30,35,36,38,39], often accompanied by compact parakeratosis in 62–94% of cases [30,35,36]. Epidermal erosion or ulceration occurs in approximately 60–70% [30,36,37,38], and pronounced acanthosis or epidermal thickening is seen in 70–95% [30,35,36,38,39]. Quantitative measurements indicate that mean epidermal thickness in invasive SCC ranges from approximately 154 to 232 µm [35,36]. The normal honeycomb architecture is lost in over 90% of SCCs [30,35,36,39], replaced by disarranged layering and focal irregular protrusions. Dyskeratotic keratinocytes are identified in roughly three-quarters of lesions [30,32,35,38,39], and atypical nuclei in more than 90% [35]. Tumour budding occurs in about 45% of invasive SCCs [32,35,39], while broad strands of atypical keratinocytes extending into the papillary dermis are seen in 29–63% of cases [30,32,35]. Keratin pearls, reflecting keratinising nests, were variably reported [30,32,35,36]. The DEJ is disrupted or non-visible in invasive SCC, with reported rates ranging widely—approximately 43% to 81%—across different series, reflecting heterogeneity in lesion characteristics and imaging conditions [30,32,35,36,39]. Vascular alterations—dilated linear vessels in 55–80% [30,35,36,38,39] and glomerular vessels in 20–80% [30,35,36]—reflect neoangiogenesis and inflammation, while dermal elastosis or collagen disorganisation appears in up to 75%, albeit with wide variability [30,32,35,36].

Table 3 provides the primary LC-OCT imaging features of keratinocytic lesions, organised by epidermal, structural, and vascular/dermal characteristics.

### 3.2. Workflow Optimisation for Keratinocytic Lesions

The LC-OCT workflow for keratinocytic lesions begins with gentle cleansing of the lesion and the application of a single drop of paraffin oil to improve optical coupling and suppress parasitic reflections [33,35]. Using the integrated macroscopic camera—and, when available, an external dermoscopic camera—the operator precisely centres the field of view before placing the probe so that the skin adheres completely to the glass window, while minimal pressure is maintained to avoid distortion of native morphology and to achieve maximal imaging depth through hyperkeratotic epidermis [38].

**Figure 3:**
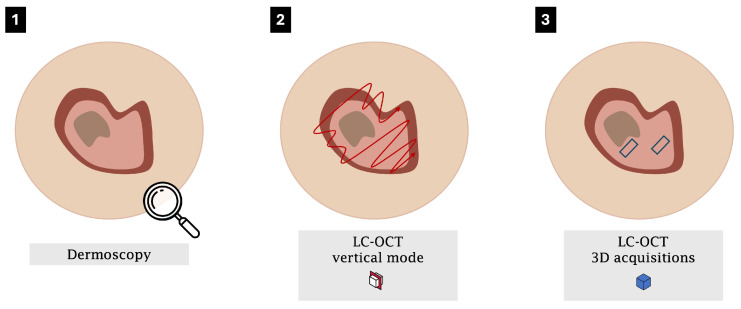
Sequential steps in the LC-OCT workflow for keratinocytic lesions: (**1**) dermoscopic localisation of the target area; (**2**) continuous vertical video sweep for comprehensive lesion coverage and assessment of the dermo-epidermal junction, avoiding the crusty part; and (**3**) acquisition of 3D volumetric stacks for depth-resolved, en-face structural visualisation, with multiple stacks recommended for lesions exhibiting intralesional heterogeneity.

Once the probe is stabilised, the acquisition of static vertical images may be replaced by a continuous vertical video sweep across the entire lesion [38], providing a cost-effective approach that delivers both a representative vertical cross-section and the dynamic benefits of full lesion coverage, minimisation of sampling errors and enhanced detection of DEJ disruption or aggressive foci. Volumetric 3D acquisitions are then used to capture true en-face views [30,31,32,34,36,38,39]—complete with depth-resolved structural information and superior image quality—so that each stack inherently includes all horizontal criteria (Figure 3). In practice, a single 3D volume often suffices for relatively homogeneous actinic keratoses, whereas lesions with marked intralesional heterogeneity (e.g., BD or SCC) warrant multiple 3D stacks—ideally two to four—sampled from central, peripheral and focally hyperkeratotic areas to ensure comprehensive architectural documentation and robust histopathological correlation. This workflow is specifically designed to account for intralesional heterogeneity and to help localise diagnostically relevant areas within a lesion, thereby improving overall diagnostic confidence.

Real-time quality monitoring allows immediate adjustment of probe pressure or position, while integrated dermoscopic guidance and mechanical stabilisation help minimise motion artefacts and maintain high contrast. Surface crusts, thick scale or ulceration—particularly common in invasive lesions—can markedly degrade signal [35,36]; therefore, three-dimensional stacks and any en-face reconstructions should be recorded at the periphery or after gentle debridement when clinically acceptable. Brief vertical videos that include adjacent, clinically normal epidermis are also valuable for visualising progressive architectural change and highlighting subtle DEJ disruption.

For research-grade studies, optional ruler-based measurements—such as epidermal thickness—may be added to correlate LC-OCT findings with histology [28,34]. With an isotropic resolution of roughly 1 µm and a penetration depth of up to 500 µm, this multimodal protocol—flexibly adjusted in the number of acquisitions to match lesion complexity—maximises the capture of tumour heterogeneity and supports clinical decision-making across the entire spectrum of keratinocytic lesions.

## 4. Discussion

This qualitative systematic review synthesised findings from eleven studies on the use of LC-OCT to diagnose AK, BD, and SCC. Across the included studies, LC-OCT consistently demonstrated enhanced visualisation of morphological features at both cellular and architectural levels, surpassing traditional clinical examination alone. LC-OCT is especially useful in lesions that are clinically or dermoscopically difficult to differentiate, as it provides in-vivo, depth-resolved information on the level of keratinocytic atypia, the status of the dermo-epidermal junction and early stromal invasion. These observations underscore the potential of LC-OCT as a non-invasive diagnostic tool that may reduce the need for biopsies and improve early detection.

Nevertheless, several limitations restrict the strength of these conclusions. The included studies varied considerably in design, sample size and LC-OCT systems, preventing direct comparison and precluding robust quantitative analyses. Because of this methodological heterogeneity and the predominantly narrative nature of the available evidence, CERQual was applied qualitatively, as the underlying data did not support the meaningful use of numerical scoring or inter-rater reliability metrics, nor the calculation of pooled diagnostic performance measures. In addition, heterogeneity related to device type, acquisition mode, lesion spectrum and operator expertise could not be quantified because these variables were inconsistently reported across studies and were therefore addressed qualitatively. Together, these features create a structural risk of upward bias in the perceived diagnostic accuracy of LC-OCT for keratinocytic lesions. The absence of standardised acquisition and interpretation protocols further limits comparability across studies, and publication bias cannot be excluded, particularly in small monocentric cohorts. Finally, the relatively limited number of eligible studies restricts generalisability.

An optimised LC-OCT workflow combines gentle lesion cleansing, paraffin-oil coupling, precise macroscopic and dermoscopic guidance under minimal probe pressure, real-time quality monitoring and mechanical stabilisation. We propose the systematic acquisition of continuous vertical video sweeps combined with depth-resolved 3D volumetric stacks to ensure comprehensive lesion coverage and robust architectural documentation, synthesising and expanding on the collected studies. Brief vertical sweeps including adjacent normal epidermis and optional ruler-based measurements further enhance detection of junctional disruption and correlation with histopathology. Systematic implementation of this flexible, multimodal workflow promises greater standardisation, reproducibility and ultimately improved clinical utility of LC-OCT for keratinocytic lesions.

Future research should focus on larger, multicentre studies employing uniform protocols to validate the diagnostic accuracy and cost-effectiveness of LC-OCT. Such efforts would facilitate robust assessments of key metrics, including sensitivity, specificity, and inter-rater reliability, and clarify the role of the modality in routine clinical practice. Priority research questions include the external validation of LC-OCT diagnostic criteria across centres, the establishment of reproducible interpretive thresholds, prospective evaluation of performance within real-world clinical workflows, and formal cost-effectiveness analyses quantifying biopsy reduction. Additionally, the integration of artificial intelligence (AI)–based image analysis, which has already demonstrated utility in diagnosing BCC, could further improve diagnostic accuracy, reproducibility, and efficiency for AK, BD, and SCC. By emphasising methodological rigour and standardised approaches, the field can fully harness the potential of LC-OCT for high-precision, non-invasive skin cancer diagnosis.

## 5. Conclusions

LC-OCT enables non-invasive, depth-resolved imaging of AK, BD and invasive SCC, demonstrating strong concordance with histopathology and substantially reducing the need for diagnostic biopsies. Clinically, its use may enhance diagnostic confidence, accelerate decision-making and inform personalised management pathways with minimal procedural morbidity. To ensure reproducibility and comparability, the adoption of harmonised acquisition protocols is imperative. Future prospective multicentre studies must employ fixed parameters to rigorously quantify diagnostic performance. Parallel development of AI-driven image-analysis algorithms will further mitigate observer variability and streamline interpretation. Consensus-based guidelines, integrating technical specifications and interpretive criteria, are essential for the widespread clinical implementation of LC-OCT. Such coordinated efforts will underpin its transition from specialised research tool to routine modality.

## Figures and Tables

**Table 1:** Characteristics of included studies.

Study	Study Design	Country	Methodology	Item Generation Method (e.g., Instrument Used to Measure Items)	Number of Patients and Lesions	Total Group Characteristics (Age, Gender, Type/Location)
Lacarrubba et al., 2023 [28]	Prospective pilot study	ITA	Ten immunocompetent patients with Olsen I actinic keratosis were enrolled; tirbanibulin 1% ointment was applied once daily for 5 consecutive days to a 25-cm^2^ area containing 4–8 AKs; clinical and LC-OCT evaluations were performed at baseline, Day 8, and Day 57 to monitor treatment response; histopathology confirmed diagnosis.	LC-OCT (DeepLive™, DAMAE Medical, Paris, France) in vertical and horizontal modes	Patients (*n* = 10) AK lesions (*n* = 55)	2 females (20%) and 8 males (80%); Mean age: 74 years (range: 62–85); Lesion locations: Scalp (80%) and face (20%).
Cianotti et al., 2023 [30]	Retrospective multicenter study	BEL and ITA	Retrospective collection of SCC and AK lesions that were imaged with both RCM and LC-OCT before surgery; three blinded observers evaluated predefined LC-OCT criteria; histopathology served as the gold standard; statistical analyses included descriptive analysis, proportion tests, and Gwet’s AC1 for agreement.	LC-OCT (CE-marked prototype, DAMAE Medical, Paris, France); ≥4 images and 1 video per lesion. RCM: VivaScope 3000 (MAVIG GmbH, Munich, Germany) ≥1 image of different depths and 1 stack per lesion	Patients (*n* = 52) Histologically proven tumors (*n* = 52): −33 SCCs (23 in situ and 10 invasive) −19 AKs	26 females (50%) and 26 males (50%); Mean age: 66.6 ± 13.7 years; SCC locations: Head/neck (54.5%), upper extremities (24.2%), trunk (12.1%), lower extremities (9.1%); AK locations: Head/neck (57.9%), upper extremities (26.3%), trunk (10.5%), lower extremities (5.3%).
Pathak et al., 2024 [31]	Descriptive observational study	USA	LC-OCT imaging of AK lesions before cryotherapy with re-evaluation at 4-week follow-up; an intention-to-treat analysis was conducted.	LC-OCT (DeepLive™, DAMAE Medical, Paris, France)	Patients (*n* = 6) Total lesions (*n* = 8)	4 females and 2 males; Mean age: 69.5 years; Lesion locations: upper arm, chest, cheek, hand, forehead, and scalp.
Jacobsen et al., 2024 [32]	Interobserver agreement study	DNK; NLD; SWE	Six evaluators blinded to histopathology independently assessed 75 LC-OCT images for the presence/absence of 10 predefined markers, also reporting confidence and artifact impact; interobserver agreement was determined using Conger’s kappa coefficient.	LC-OCT (DeepLive™, DAMAE Medical, Paris, France); high-resolution video acquisitions (cross-section and en-face views)	Total lesions (*n* = 75) 21 SCC 21 BCC (including 6 superficial and 15 nodular) 12 BD 21 AK (including 6 grade I, 9 grade II, and 6 grade III)	NR
Donelli et al., 2023 [33]	Prospective observational monocentric study	BEL; ESP; FRA; ITA	Included all cutaneous lesions with uncertain clinical/dermoscopic diagnoses of possible malignant skin tumors (excluding lesions on the eyelid margin, internal cantus, and upper eyelid); dermoscopic and LC-OCT diagnoses were entered prospectively into software; histopathology was used as the gold standard.	LC-OCT (DeepLive™, DAMAE Medical, Paris, France) providing vertical, horizontal, and 3D images. Dermoscopy using VivaCam D200 and Dermlite DL200 Hybrid handheld	Lesions analyzed: 1481 total; final analysis included 466 lesions (312 excised, 149 biopsied, 5 followed up). BCC (*n* = 152), AK (*n* = 20), Bowen’s disease (*n* = 14), SCC (*n* = 22), intradermal nevi (*n* = 4), seborrheic keratosis (*n* = 14), sebaceous hyperplasia (*n* = 1), inflammatory lesions (*n* = 15), other lesions (*n* = 70)	NR
Ruini et al., 2021 (second) [34]	Pilot observational study	DEU	Fifty facial AKs clinically suspected were imaged with LC-OCT (vertical mode) before biopsy (within 0–7 days); observers classified the basal proliferation pattern (PRO I, II, III) using LC-OCT vertical images; histopathology served as the gold standard.	LC-OCT (CE-marked prototype, DAMAE Medical, Paris, France); vertical mode	Patients (*n* = 43) Histopathologically confirmed AK lesions (*n* = 50); 5 excluded due to insufficient image quality	17 females (40%) and 26 males (60%); Mean age: 73.8 years (range: 57–86); Lesion location: face (100%).
Cianotti et al., 2021 [35]	Retrospective observational multicentre study	BEL and ITA	Lesions clinically suspected of AK or SCC were imaged with LC-OCT before surgical excision and then examined histopathologically; three blinded observers evaluated LC-OCT image quality and criteria; univariate and multivariate analyses were performed.	LC-OCT (CE-marked prototype, DAMAE Medical, Paris, France); ≥4 images and 2 videos per lesion	Patients (*n* = 158) Histologically confirmed lesions (*n* = 158): 108 SCC (62 in situ, 46 invasive) and 50 AK	83 females (52.5%) and 75 males (47.5%); Mean age: 69.8 ± 12.7 years; SCC: Head/neck (54.6%), legs (17.6%), arms (14.8%), trunk (13%); AK: Head/neck (62%), legs (14%), arms (12%), trunk (12%).
Ruini et al., 2021 (first) [36]	Prospective observational study	DEU	Clinical, dermoscopic, and LC-OCT images were prospectively collected and analyzed for lesions suspected of keratinocyte skin cancer; histopathology was used as the gold standard; exemplary lesions were additionally investigated with conventional OCT and RCM for comparison.	LC-OCT (CE-marked prototype, DAMAE Medical, Paris, France); Vertical, horizontal, and 3D modes. Dermoscopy (FotoFinder/DermoGenius) OCT: VivoSight device (Michelson Diagnostics, Orpington, UK) RCM: VivaScope 3000 camera (MAVIG GmbH, Munich, Germany)	Histopathologically confirmed lesions (*n* = 73) −46 AKs (10 hypertrophic, 5 atrophic, 3 bowenoid) −11 Bowen’s disease −16 SCCs	25 females (34%) and 48 males (66%); Mean age: 74.8 years; Lesion locations: Head/neck (52.1%), scalp (28.8%), upper limbs (9.6%), lower limbs (5.5%), trunk (2.7%), genital area (1.4%).
Lenoir et al., 2021 [37]	Case series	BEL; ITA; FRA; ESP	Lesions clinically and dermoscopically suggestive of actinic keratosis (AK) were included; AK subtypes (atrophic, hypertrophic, proliferative, acantholytic) were characterized based on LC-OCT findings; histopathology served as the gold standard for diagnostic confirmation.	LC-OCT (CE-marked prototype, DAMAE Medical, Paris, France); vertical, horizontal, and 3D modes	Histopathologically confirmed AK lesions (*n* = 16)	NR
Di Stefani et al., 2023 [38]	Retrospective observational study	ITA	Patients with biopsy-proven equivocal eyelid skin lesions underwent in vivo LC-OCT imaging before surgical excision; images were evaluated by two investigators (with a third for discrepancies); histopathological examination was used as the gold standard.	LC-OCT (DeepLive™, DAMAE Medical, Paris, France) in vertical and horizontal modes	Patients (*n* = 51) Histopathologically confirmed lesions (*n* = 51)	28 females (55%) and 23 males (45%); Mean age: 66.4 years (range: 34–88); Lesion locations: Lower eyelid (39%), medial canthus (27%), upper eyelid (18%), lateral canthus (16%).
Verzì et al., 2024 [39]	Case series	ITA	Eyelid margin lesions with a challenging clinical appearance (onset ≤ 12 months) were imaged before surgical excision; histopathological examination was used as the gold standard.	LC-OCT (DeepLive™, DAMAE Medical, Paris, France)	Patients (*n* = 28) Histopathologically confirmed lesions (*n* = 31)	13 females (46%) and 15 males (54%); Mean age: 64.7 years (range: 44–87); Lesion locations: Upper eyelid (13 cases) and lower eyelid (18 cases).

Abbreviations: BEL, Belgium; DNK, Denmark; FRA, France; DEU, Germany; ITA, Italy; NLD, Netherlands; SWE, Sweden; USA, United States of America; OCT, Optical coherence tomography; RCM, Reflectance confocal microscopy; BCC, Basal cell carcinoma; BD, Bowen’s disease. NR, not reported.

**Table 2:** CERQual confidence assessment.

Keratinocytic Lesions	Studies That Contributed	Methodological Limitations (No/Minor/Moderate/Serious Limitations)	Coherence	Adequacy	Assessment of Confidence (High/Moderate/Low/Very Low)
Actinic keratosis	[28,30,31,32,33,34,35,36,37,38]	Minor (consistent reporting across multiple studies with histopathological validation)	High (features align strongly across studies with consistent percentages and descriptions)	Rich (detailed quantitative measurements and qualitative descriptions from multiple studies)	High
Bowen’s Disease	[32,35,36]	Moderate (fewer studies focusing specifically on Bowen’s)	High (features align between studies despite varying terminology)	Moderate (detailed descriptions but from limited number of studies)	Moderate
Invasive SCC	[30,32,35,36,37,38,39]	Minor (consistent reporting with histopathological validation across multiple studies)	High (features align strongly across studies with complementary descriptions)	Rich (detailed quantitative measurements with multiple supporting studies)	High

**Table 3:** LC-OCT imaging features.

Keratinocytic Lesions	Studies That Contributed	Epidermal	Structural	Vascular/Dermal
Actinic keratosis	[28,30,31,32,33,34,35,36,37,38]	Hyperkeratosis (42–100%) Parakeratosis (64–83%) Acanthosis (71–76%) Keratinocytic atypia (82–100%)	Mean epidermal thickness 105–126.2 μm Well-preserved/visible DEJ (48–70%) Architectural disorganisation (77.8–97%) Tumour budding (45–58%)	Dilated vessels (57.1–89.5%) Elastosis/collagen alterations (26.2–61%)
Bowen’s Disease	[32,35,36]	Hyperkeratosis (69.4–100%) Parakeratosis (63–100%) Keratinocytic atypia (73.3–100%)	Mean epidermal thickness 141–168.5 μm DEJ well-defined (24–80%), Bowenoid pattern (90%) Architectural disorganisation (>90%) Tumour budding (33.3–49%) Broad strands (9.7%)	Dilated vessels (44–70%) Glomerular vessels (20–61.9%) Elastosis/collagen alterations (10–70%)
Invasive SCC	[30,32,35,36,37,38,39]	Hyperkeratosis (72.7–100%) Parakeratosis (62–93.8%) Acanthosis (75–93.8%) Marked keratinocytic atypia (70–84%) Atypical nuclei (95%) Erosion/ulceration (63–68%)	Mean epidermal thickness 154–232 μm DEJ well-defined (18.8–57%) Architectural disorganisation (93–100%) Tumour budding (44–45%) Broad strands (29–63%)	Dilated vessels (55–78.6%) Glomerular vessels (21.4–80%) Elastosis/collagen alterations (7.4–75%) Keratin pearls (0–81%)

## Data Availability

The original contributions presented in this study are included in the article/Supplementary Material. Further inquiries can be directed to the corresponding author.

## Supplementary Materials

The following supporting information can be downloaded at: https://www.mdpi.com/article/10.3390/diagnostics15212746/s1, Table S1: Complete search strategies for PubMed, Embase, and Scopus, Table S2: PRISMA 2020 Checklist.
