# Supplementary material for: Systematic Review of Line-Field Confocal Optical Coherence Tomography for Diagnosing Pre-Malignant and Malignant Keratinocytic Lesions: Optimising the Workflow
Source: Diagnostics (Basel). 2025 Oct 29;15(21):2746. doi: 10.3390/diagnostics15212746 (PMC12609820; doi:10.3390/diagnostics15212746)
Supplement: Supplementary file 1 [file diagnostics-15-02746-s001.zip › Table S1.pdf]

Table S1: Complete search strategies for PubMed, Embase, and Scopus.

Database	Concepts	Detailed Terms	Combined Strategy	Filters Applied
PubMed	Concept 1: LC-OCT<br>Concept 2: Actinic Keratosis (AK)<br>Concept 3: Squamous Cell Carcinoma (SCC)	"Line-field confocal optical coherence tomography"[TIAB] OR "LC-OCT"[TIAB]<br><br>AK terms: Actinic Keratosis[MeSH], "AK"[TIAB], "Solar Keratosis", "Precancerous skin lesion", "Sun damage", hypertrophic, atrophic, bowenoid, acantholytic, epidermolytic, lichenoid, pigmented<br><br>SCC terms: Squamous Cell Carcinoma[MeSH], "SCC"[TIAB], "Cutaneous SCC", "Bowen's Disease", "Squamous Cell Carcinoma in situ", "Intraepidermal Carcinoma", squamous cell carcinoma in situ, Bowen disease, acantholytic, adenoid, pseudoglandular, clear cell, sarcomatoid, spindle cell, desmoplastic, keratoacanthoma, verrucous carcinoma	(Concept 1 AND Concept 2) OR (Concept 1 AND Concept 3) OR (Concept 1 AND Concept 2 AND Concept 3)<br>NOT (Animals[MeSH] NOT Humans[MeSH])	2018–2024; English; Humans only; Excluded preprints (n=24)

Database	Concepts	Detailed Terms	Combined Strategy	Filters Applied
Embase	Concept 1: LC-OCT<br>Concept 2: AK<br>Concept 3: SCC	Concept 1: ("Line-field confocal optical coherence tomography" OR "LC-OCT").ti,ab.<br><br>Concept 2: exp actinic keratosis/ OR (AK, "Solar Keratosis", "Precancerous skin lesion", "Sun damage", hypertrophic, atrophic, bowenoid, acantholytic, epidermolytic, lichenoid, pigmented).ti,ab.<br><br>Concept 3: exp squamous cell carcinoma/ OR (SCC, "Cutaneous SCC", "Bowen's Disease", "Squamous Cell Carcinoma in situ", "Intraepidermal Carcinoma", squamous cell carcinoma in situ, Bowen disease, acantholytic, adenoid, pseudoglandular, "clear cell", sarcomatoid, "spindle cell", desmoplastic, keratoacanthoma, "verrucous carcinoma").ti,ab.	(#1 AND #2) OR (#1 AND #3) OR (#1 AND #2 AND #3)	2018–2024; English; Humans only; Articles and reviews only; Excluded preprints; Excluded MEDLINE records (n=5, duplicates reduced to n=20)

Database	Concepts	Detailed Terms	Combined Strategy	Filters Applied
Scopus	Concept 1: LC-OCT<br>Concept 2: AK<br>Concept 3: SCC	Concept 1: TITLE-ABS-KEY("Line-field confocal optical coherence tomography" OR "LC-OCT")<br><br>Concept 2: TITLE-ABS-KEY("Actinic Keratosis" OR "AK" OR "Solar Keratosis" OR "Precancerous skin lesion" OR "Sun damage" OR hypertrophic OR atrophic OR bowenoid OR acantholytic OR epidermolytic OR lichenoid OR pigmented)<br><br>Concept 3: TITLE-ABS-KEY("Squamous Cell Carcinoma" OR "SCC" OR "Cutaneous SCC" OR "Bowen's Disease" OR "Squamous Cell Carcinoma in situ" OR "Intraepidermal Carcinoma" OR "squamous cell carcinoma in situ" OR "Bowen disease" OR acantholytic OR adenoid OR pseudoglandular OR "clear cell" OR sarcomatoid OR "spindle cell" OR desmoplastic OR keratoacanthoma OR "verrucous carcinoma")	(Concept 1 AND Concept 2) OR (Concept 1 AND Concept 3) OR (Concept 1 AND Concept 2 AND Concept 3)	PUBYEAR > 2017 AND PUBYEAR < 2025; English; Journals only; NOT INDEX(medline)
